# Supplementary material for: A comparative test of inequity aversion in domestic dogs (Canis familiaris) and dingoes (Canis dingo)
Source: PLoS One. 2021 Sep 22;16(9):e0255885. doi: 10.1371/journal.pone.0255885 (PMC8457503; doi:10.1371/journal.pone.0255885)

Supplementary Materials for *A comparative test of inequity aversion in domestic dogs (Canis familiaris) and dingoes (Canis dingo)*

Table of Contents

Table S1. Domestic dog subject information 2

Table S2. Dingo dog subject information 4

Figure S1. Boxplot figure of approach data by species 5

Figure S2. Boxplot figure of reaction time data by species 6

Figure S3. Boxplot figure of referencing behaviour by species 7

Table S1. Breed, sex and condition information for dog subjects

Subject Name	Breed	Sex	Control Group	Confederate Partner	Day 1 Condition	Day 2 Condition
Angus	Irish Wolfhound	M	NSNR	Taaka	INEQ	NSNR
Annie	Pointer/Hound Mix	F	SNR	Stringer	SNR	INEQ
Ariel	Bernese Mountain Dog	F	SNR	Stringer	SNR	INEQ
Belichick	Puggle	F	NSNR	Stringer	NSNR	INEQ
Bergamot	Standard Poodle	F	NSC2	Taaka	INEQ	NSC2
Byron	Beagle	M	NSNR	Taaka	NSNR	INEQ
Chloe	Labradorrador	F	NSNR	Taaka	INEQ	NSNR
Cleo	Newfoundland	F	NSC2	Taaka	INEQ	NSC2
Clover	Goldendoodle	F	SNR	Stringer	SNR	INEQ
Clytie	Pembroke Welsh Corgi	F	NSNR	Taaka	INEQ	NSNR
Curley	Mix	M	SNR	Stringer	SNR	INEQ
Daisy1	Norwich Terrier	F	NSC1	Taaka	INEQ	NSC1
Daisy3	Greyhound/Labrador Mix	F	SNR	Stringer	INEQ	SNR
Danko	Black Mouth Cur	M	NSC1	Taaka	NSC1	INEQ
Dayton	Labrador/Golden Poodle	M	NSC2	Taaka	NSC2	INEQ
Diego	Portuguese Water Dog	M	SNR	Taaka	INEQ	SNR
Duke	Husky/Boxer	M	NSC1	Stringer	NSC1	INEQ
Ebony	Labrador/Beagle	F	NSC1	Taaka	NSC1	INEQ
Elzi	Shepherd Mix	F	NSC1	Taaka	INEQ	NSC1
Georgie	Labrador	M	NSC2	Taaka	NSC2	INEQ
Gilligan	Labrador Mix	M	NSC2	Taaka	NSC2	INEQ
Gracie	Dachshund	F	NSC1	Stringer	INEQ	NSC1
Gretchen	German Shepherd	F	NSC1	Stringer	INEQ	NSC1
Gromit	Border Collie	M	NSC1	Taaka	NSC1	INEQ
Gus	Cockapoo	M	NSC1	Stringer	INEQ	NSC1
Harry	Tibetan Terrier	M	SNR	Taaka	INEQ	SNR
Hawkeye	Border Collie	M	SNR	Stringer	SNR	INEQ
Honey	Golden Retriever	F	NSNR	Stringer	INEQ	NSNR
Hugo	Border Terrier	M	NSC1	Taaka	NSC1	INEQ
Jake	Puggle	M	NSC2	Stringer	INEQ	NSC2
Juno	Siberian Husky	F	SNR	Stringer	SNR	INEQ
Kapakahi	Foxhound	F	NSC1	Taaka	INEQ	NSC1
Kyler	Pembroke Welsh Corgi	M	NSC2	Taaka	NSC2	INEQ
Kyra	Rhodesian Ridgeback Mix	F	SNR	Stringer	INEQ	SNR
Lily	Bernese Mountain Dog	F	NSNR	Taaka	INEQ	NSNR
Lindy	Golden Retriever	F	NSNR	Stringer	INEQ	NSNR
Loki	Shiba Inu Mix	F	SNR	Taaka	INEQ	SNR
Louis	Mix	M	NSNR	Stringer	NSNR	INEQ
Lucy	Shepherd Mix	F	NSC2	Taaka	NSC2	INEQ
Luna1	Labrador Mix	F	NSC1	Taaka	INEQ	NSC1
Luna2	Border Collie	F	NSNR	Taaka	INEQ	NSNR
Mac	Border Collie	M	NSC2	Stringer	INEQ	NSC2
Mackenzie	Scottish Terrier	M	SNR	Stringer	INEQ	SNR
Maggie1	Cocker Spaniel	F	NSC1	Stringer	NSC1	INEQ
Maggie2	Labrador	F	NSC1	Taaka	INEQ	NSC1
Mai	Pit Bull Mix	F	NSC2	Taaka	NSC2	INEQ
Nunu	Great Pyrenees	F	SNR	Stringer	INEQ	SNR
Obikai	Pomeranian	M	SNR	Stringer	SNR	INEQ
Olivia	Cocker Spaniel	F	SNR	Stringer	INEQ	SNR

Omni	Rat Terrier	F	SNR	Stringer	INEQ	SNR
Oonah	Malamute	F	NSC2	Taaka	INEQ	NSC2
Papita	Papillion	F	NSNR	Taaka	INEQ	NSNR
Piper	Mutt/Shelter	F	NSNR	Taaka	INEQ	NSNR
Ponder	Cockapoo	M	NSNR	Taaka	NSNR	INEQ
Riley	Springer Spaniel	M	NSNR	Stringer	INEQ	NSNR
Rose	Border Terrier	F	NSNR	Taaka	INEQ	NSNR
Rosie1	Terrier Mix	F	NSC1	Taaka	INEQ	NSC1
Rosie2	Carolina Dog	F	NSC2	Taaka	INEQ	NSC2
Roxanne	German Shepherd	F	NSC2	Taaka	NSC2	INEQ
Ruby	Labrador/Terrier Mix	F	SNR	Stringer	SNR	INEQ
Sammie	Chow Mix	F	NSC2	Taaka	NSC2	INEQ
Sass	Labrador	F	SNR	Stringer	SNR	INEQ
Sioux	Australian Shepherd	F	NSNR	Taaka	NSNR	INEQ
Slipper	Labrador/Hound Mix	M	NSC2	Taaka	INEQ	NSC2
Snickers	Labradoodle	F	SNR	Stringer	SNR	INEQ
Spec	Jack Russell	M	NSNR	Taaka	NSNR	INEQ
Spot	Parson Russell Terrier	M	NSC2	Stringer	NSC2	INEQ
Sweetums	Bulldog	F	NSC1	Taaka	NSC1	INEQ
Sydney	Mix	F	NSC1	Taaka	NSC1	INEQ
Tahdy	Portuguese Water Dog	F	NSNR	Stringer	NSNR	INEQ
Tessie	German Shepherd	F	NSNR	Taaka	NSNR	INEQ
Nezumi	German Shepherd	F	NSC1	Taaka	NSC1	INEQ

Table S2. Sex and condition information for dingo subjects

Subject Name	Sex	Confederate	Day 1 Condition	Day 2 Condition	Day 3 Condition	Day 4 Condition	Day 5 Condition
AyJay	F	Deedee	INEQ	NSC2	NSC1	NSNR	SNR
Fanta	F	Deedee	NSNR	NSC2	SNR	INEQ	NSC1
Minka	F	Deedee	NSNR	SNR	NSC2	NSC1	INEQ
Opal	F	Deedee	SNR	NSC1	NSNR	INEQ	NSC2
Petal	F	Deedee	SNR	NSNR	NSC1	NSC2	INEQ
Rosie	F	Deedee	NSC1	INEQ	SNR	NSC2	NSNR
Snapple	M	Deedee	SNR	NSNR	NSC1	NSC2	INEQ
Tilly	F	Deedee	NSC2	INEQ	NSNR	NSC1	SNR
Wirra	F	Deedee	NSC2	NSNR	INEQ	SNR	NSC1
Yaouk	M	Deedee	INEQ	NSC1	NSC2	SNR	NSNR
Yoori	M	Deedee	NSC1	SNR	INEQ	NSNR	NSC2

Figure S1. Number of times dogs and dingoes approached in baseline (top row: both subject and partner received a reward) and test sessions (bottom row: subject received nothing) across five conditions. Domestic dogs were tested in a between-subject design in which each dog received one session where condition was Inequity (gray bars) and one session where condition was one of four controls. Dingoes were tested in a within-subject design in which all subjects received all five conditions (Inequity, SNR, NSC1, NSC2, NSNR). Black lines bisecting each box show median values, boxes represent inter-quartile ranges, lines extending from boxes indicate calculated minimum and maximum values and black circles indicate outliers.

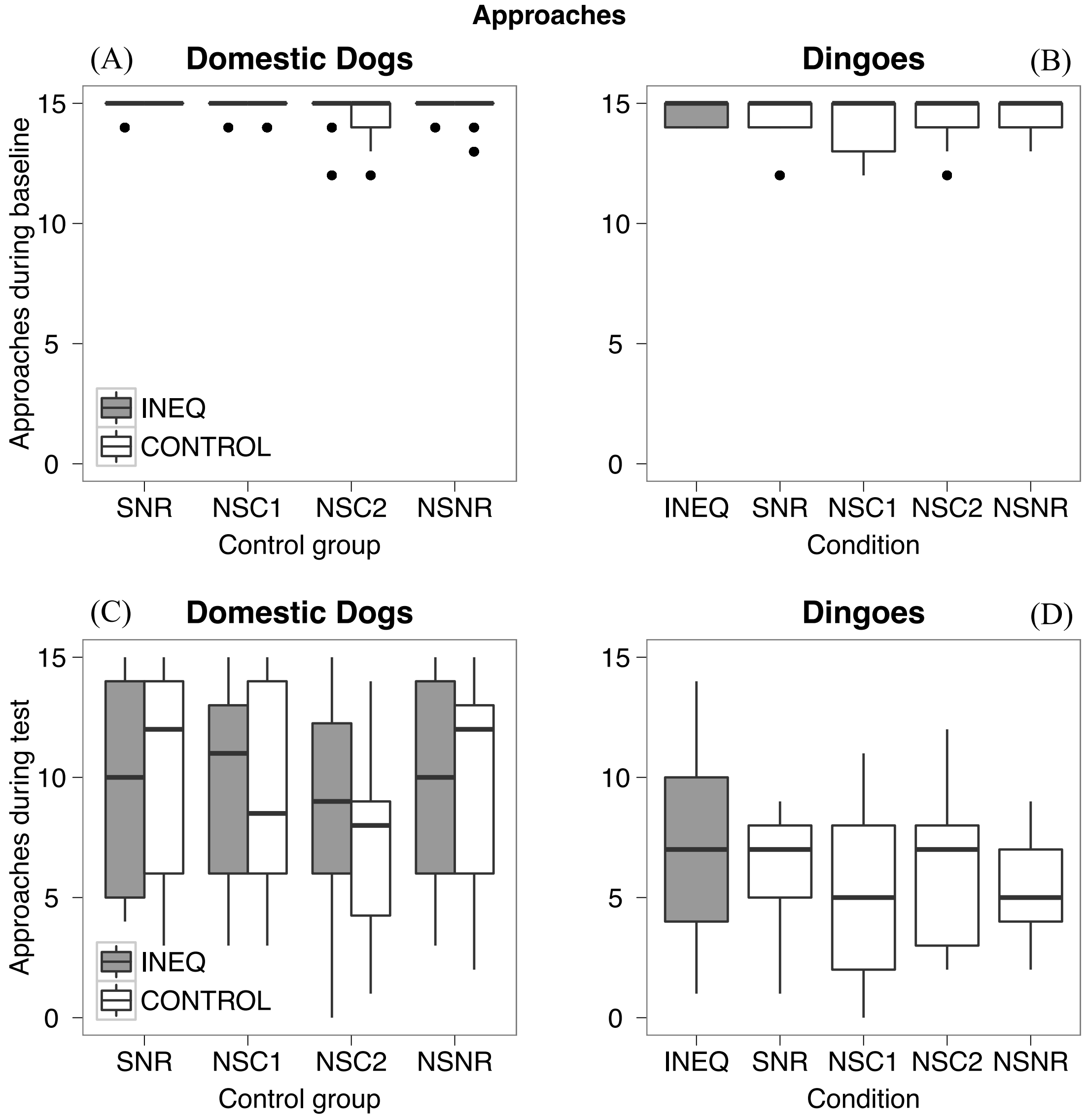

Figure S2. Time it took dogs and dingoes to approach (reaction time) in test sessions (subject received nothing) across five conditions. Domestic dogs were tested in a between-subject design in which each dog received one session where condition was Inequity (gray bars) and one session where condition was one of four controls. Dingoes were tested in a within-subject design in which all subjects received all five conditions (Inequity, SNR, NSC1, NSC2, NSNR). Black lines bisecting each box show median values, boxes represent inter-quartile ranges, lines extending from boxes indicate calculated minimum and maximum values and black circles indicate outliers.

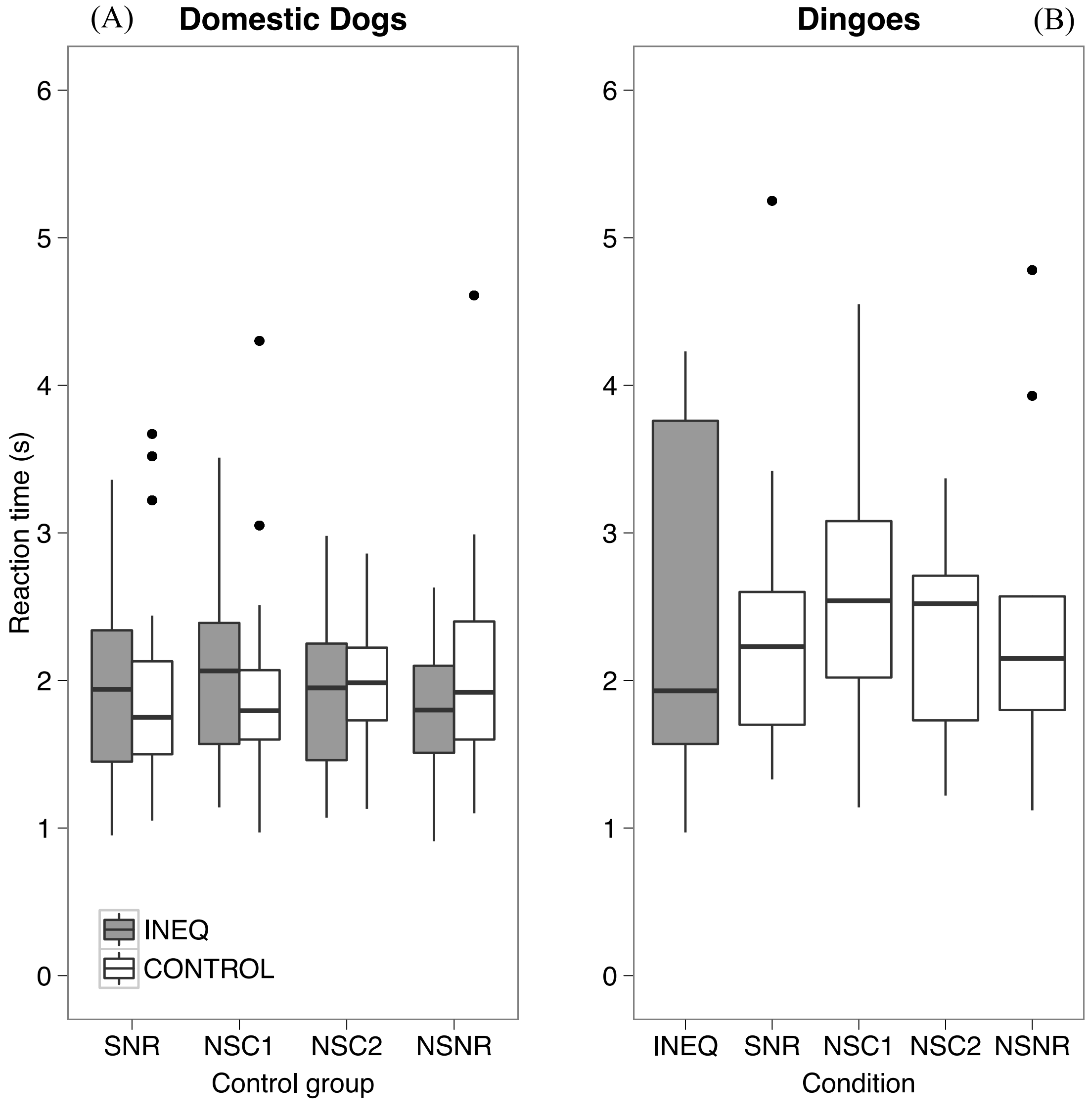

Figure S3. Number times dogs and dingoes looked at the experimenter (top row) or handler (bottom row) in test sessions (subject received nothing) across five conditions. Domestic dogs were tested in a between-subject design in which each dog received one session where condition was Inequity (gray bars) and one session where condition was one of four controls. Dingoes were tested in a within-subject design in which all subjects received all five conditions (Inequity, SNR, NSC1, NSC2, NSNR). Black lines bisecting each box show median values, boxes represent inter-quartile ranges, lines extending from boxes indicate calculated minimum and maximum values and black circles indicate outliers.

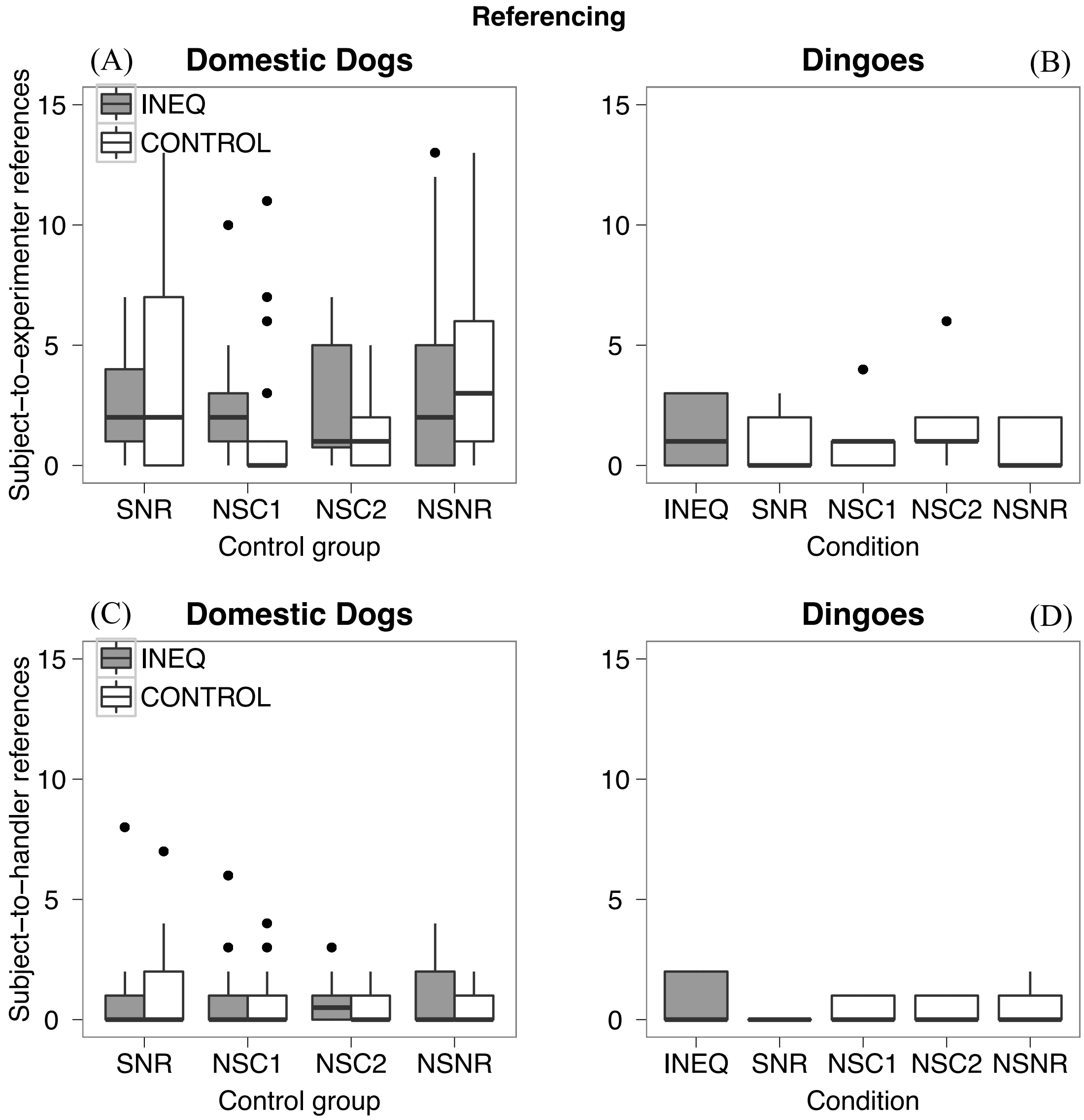

Supplement: S1 File — (PDF) [file pone.0255885.s001.pdf]
