# Supplementary material for: A comparative test of inequity aversion in domestic dogs (Canis familiaris) and dingoes (Canis dingo)
Source: PLoS One. 2021 Sep 22;16(9):e0255885. doi: 10.1371/journal.pone.0255885 (PMC8457503; doi:10.1371/journal.pone.0255885)
Supplement: Attachment — Submitted filename: Inequity Aversion Dogs and Dingoes_Response Letter.pdf [file pone.0255885.s006.pdf]

JOURNAL REQUIREMENTS

1. Please ensure that your manuscript meets PLOS ONE's style requirements, including those for file naming.

The MS now meets the PLOS ONE style requirements.

2. In your Methods section, please provide additional details regarding participant consent from the owners of the animals. In the ethics statement in the Methods and online submission information, please ensure that you have specified (i) whether consent was informed and (ii) what type you obtained (for instance, written or verbal). If the need for consent was waived by the ethics committee, please include this information.

I have provided additional details regarding participant consent.

3. We note that you have stated that you will provide repository information for your data at acceptance. Should your manuscript be accepted for publication, we will hold it until you provide the relevant accession numbers or DOIs necessary to access your data. If you wish to make changes to your Data Availability statement, please describe these changes in your cover letter and we will update your Data Availability statement to reflect the information you provide.

If the article is accepted, I will upload data and R code (as per Reviewers' request) to OSF. For now, I have provided both data and code as supplemental material.

4. Please include captions for your Supporting Information files at the end of your manuscript, and update any in-text citations to match accordingly. Please see our Supporting Information guidelines for more information: http://journals.plos.org/plosone/s/supporting-information

Captions are now included at the end of the MS.

5. We note that Figure 1 includes images of participants in the study.

As per the PLOS ONE policy (http://journals.plos.org/plosone/s/submission-guidelines#loc-human-subjects-research) on papers that include identifying, or potentially identifying, information, the individual(s) or parent(s)/guardian(s) must be informed of the terms of the PLOS open-access (CC-BY) license and provide specific permission for publication of these details under the terms of this license.

a. Please download the Consent Form for Publication in a PLOS Journal (http://journals.plos.org/plosone/s/file?id=8ce6/plos-consent-form-english.pdf). The signed consent form should not be submitted with the manuscript, but should be securely filed in the individual's case notes.

b. Please amend the methods section and ethics statement of the manuscript to explicitly state that the patient/participant has provided consent for publication: "The individual in this manuscript has given written informed consent (as outlined in PLOS consent form) to publish these case details".

If you are unable to obtain consent from the subject of the photograph, you will need to remove the figure and any other textual identifying information or case descriptions for these individuals.

I am no longer able to contact the people who appear in the photo with the dogs and dingoes. For all people except me (I am in the center of both images), I have added a black box over their faces to de-identify them. I have completed and attached the consent form.

REVIEWER #1

Comment R1.1.
This was a very clearly written and well balanced article. I found it very easy to read and follow and I especially enjoyed the inclusion of dingoes and the comparisons drawn with domestic dogs. I think this article will be well received and I have only a few minor questions/comments for clarity.

Response R1.1.
Thank you for your positive evaluation of the article and for your helpful suggestions for how to improve it further.

Comment R1.2.
I believe this is a single author article and yet sometimes the third person voice is used
(e.g., line 179 “Our third measure…”) – I suggest just using first person singular

Response R1.2.
Thank you for noting this. I have changed this throughout.

Comment R1.3.
Line 267 “ Regardless of condition, subjects never received a treat for approaching during test trials.” Approaching what?

Response R1.3.
I have clarified that this referred to subjects approaching the plate.

Comment R1.4.
Line 268 “Test trials were always preceded by baseline trials in order to control for the possibility that subjects would lose motivation over the course of a session: because the non-rewarded part of the session always occurred after the rewarded part of the session, the potential effects of loss of motivation were consistent across all conditions.” But do you think this may have also buffered any effects of inequity?

Response R1.4.
Thank you for raising this. This is a good point and one I now raise in a new section in the discussion.

Comment R1.5
Table 1 please state explicitly in each of the descriptions what and how many rewards were put on each plate in each condition. If the reward count was consistent across conditions, perhaps this can go in the caption.

Response R1.5.
In all cases, a single unit of the food reward was placed on the plate. I have made this clear in the caption.

Comment R1.6.
Table 1 in the non-social baseline, was food also put on the second plate?

Response R1.6.
Yes, in the non-social baseline, food was put on both plates as it was in the social baselines. I have added this information to Table 1.

Comment R1.7.
Line 328 Sorry if I missed it, but how far were the dogs and dingoes (in distance) from the plates at the start of each trial?

Response R1.7.
The distance from the dogs’ taped-off rectangles to the line in the middle of the room was approximately 6 feet and 11 inches. The distance from the plate would therefore be about 6 feet (Fig. 1). I have added this information to the methods.

Comment R1.8.

Line 348 “In cases where a trial was deemed invalid during testing (e.g., due to a mistimed dog release by the handler) the trial was redone.” How often did this happen?

Response R1.8.
I have now included the information.

Comment R1.9.
Line 344 what was the inter-trial interval? i.e how long did the animals have to eat the food (if available) before being recalled to their handler?

Response R1.9.
Subjects could eat the food between their approach to the plate and the next trial. Food items were small and thus typically consumed immediately. However, this meant that subjects could, in principle, eat while they were being walked back to the handlers. Between trials, the experimenter made notes on the live coding sheets and prepped the next food items. Judging from a sample of videos, this interval was between 10s and 30s, depending, for instance, on how many live coding notes were needed for a given trial. I have added this information to the methods section.

Comment R1.10.
Line 413 this ethical approval reference is a little unclear – what institution is this associated with? And does this apply to both the USA and Australia? Furthermore, please ensure and confirm that your reporting meets the ARRIVE 2.0 guidelines (Percie du Sert et al. 2020 The ARRIVE guidelines 2.0: Updated guidelines for reporting animal research. PLoS Biol 18(7): e3000410).

Response R1.10.
Thank you for this reference. In the revised MS, I name the ethical review committee (Harvard University IACUC) that approved this study in accordance with the recommended information under item #14 of the table in the referenced MS.

Comment R1.11.
You might consider including tables S3 and S4 in the main body of your article.

Response R1.11.
Thank you for this suggestion. I have moved these to the main text. Note that I also recreated these tables with bootstrapped CIs.

Comment R1.12.
I think you should also consider providing your raw R script/code as supplementary materials to accompany your data sets.

Response R1.12.
I now provide the R code as well. Thank you for this suggestion.

REVIEWER 2

Comment R2.1.
The current study compares dogs' and dingoes' response to inequity in a novel inequity task. Subjects' approach to a plate was assessed in an inequity condition (i.e. when no food was placed on their plate but food was placed on the plate of a conspecific partner behind a transparent barrier) and compared with responses in control conditions that varied with regards to the presence of a partner and the presence of food on the partner's plate. If subjects were inequity averse, they should have approached fewer times and taken longer to approach in the inequity than in the control conditions. Overall, neither dogs nor dingoes approached fewer times or took longer to approach in the inequity condition compared with the control conditions. Although the findings in this study contradict those of other studies that demonstrate inequity aversion in dogs, the author points out some potential methodological reasons for the absence of inequity aversion.

Overall, the manuscript is well written and provides a good background to the field. The author also does a good and fair job of interpreting the results in the discussion, particularly in the light of the sizeable literature on inequity aversion in dogs that already exists and potential methodological explanations for the lack of inequity aversion. I think it is also commendable that the author tries to develop a new inequity task for use with non-human animals/dogs that is more similar to tasks used with humans.

I have some comments which I believe will help improve the manuscript and these largely relate to additional points I feel should be raised in the discussion, some of these highlighting methodological issues.

Response R2.1.
Thank you very much for your helpful feedback on this manuscript. I have addressed each of your points below and believe that these revisions have added clarity to the paper. I especially appreciated your suggestions for additions to the discussion, which I believe have led to a more thoughtful consideration of the findings.

Major comments:

Comment R2.2.
Lines 179 – 183: Although it might make sense that dogs would have a greater tendency to look at the humans, it is not clear enough to me from the introduction so far why this measure is something worthwhile to look at with regards to inequity aversion…e.g. is it a measure of inequity aversion? If there is no difference in social referencing across conditions but there is a difference in approach would this change anything about the conclusion? If there is a difference in social referencing across conditions, it is also not clear what this would mean. It seems to me that the social reference measure would not tell us much. There could be a good reason for including it and there may be a good argument for it but then I would suggest giving a stronger reason here in the introduction. Otherwise, I think the other two measures are sufficient as they give clearer answers to the questions addressed. Whether the social referencing is omitted or explained better or not changed at all is up to the author and should not change the decision regarding acceptance in my view, though excluding the referencing would lead to quite a few changes to the discussion.

Response R2.2.
Thank you for raising this. At the end of the revised introduction, I now include a statement detailing the rationale behind the inclusion of this measure. Specifically: "Social referencing was included as a measure because, in line with work using unsolvable tasks with canids (e.g., [42, 49]), subjects may treat unequal payoffs as a 'problem' that needs to be solved, in which case one would expect to see more social referencing in the experimental than control conditions."

Comment R2.3.
Line 207: The fact that there were only 3 partners in the whole study, as far as I understood, is a problem in terms of pseudoreplication. The study potentially asks whether the individuals are averse to inequity with that specific partner. Of course, we do not know whether the unfamiliarity of the partner, or the partner-specific traits, account for much of the response; therefore, it is difficult to assess the extent to which this issue explains the results. There is not much that can be done about this now but I think this point should be raised in the discussion.

Response R2.3.
This is a good point and one that I now address in a new section in the discussion.

Comment R2.4.

Lines 236 – 242: It is good that both species were tested in the type of environment that they were familiar with but the fact that these were different environments could also be a problem. I think this should also be addressed at least briefly in the discussion.

Response R2.4
This is also a good point and one that I have addressed in the new section in the discussion that I mentioned above.

Comment R2.5.
Discussion: In general, the discussion addresses a lot of very good points. One major point which is not really touched on which I would like to see discussed is whether the subjects actually paid attention to the partner and whatever else was happening on the other side of the barrier, including the differences in rewards. The results seem consistent with the idea that the dogs did not pay any attention to what was going on (apart from the indication that the presence of a partner affected some aspects of their behaviour, but one could respond naturally to the presence of a conspecific in the vicinity without paying attention specifically to the events on the other side of the transparent barrier). It would be useful to have an extra variable such as gazing at the partner or gazing at the partner's food, though I recognise this could be quite difficult to code and might not give a very clear answer. Either way, I think it would be good to include more discussion around this point about attention to the partner and the partner's rewards. See also related comments about specific points in the discussion.

Response R2.5.
I agree that this point deserved more attention and, to this end, I have added a new paragraph that focuses on this point beyond the context of subject-to-experimenter referencing (see also Responses 2.6 and 2.7).

Comment R2.6.
Lines 586 – 588: I would like to see an elaboration of these points: Why would the subjects not have understood the experimenter's role and why would they not have viewed the absence of food as a social problem? I understand these points are being raised in the context of dogs referencing the human but the answers to these questions might also provide an explanation for why no inequity aversion was observed (if dogs and dingoes are actually inequity averse).

Response R2.6.
Thank you for this suggestion. I have added a paragraph elaborating on these points.

Comment R2.7.
Lines 589 – 591: It seems this could also be an elaboration of or explanation for the points in the previous sentence, even though it is not presented as such i.e. the subjects might not have understood or paid attention to the experimenter as they were focused on searching for food. Also, they might not have viewed it as a social issue as they may have been too focused on whether there was food on their own side. Consider including this as a possible explanation.

Response R2.7.
This is also a good point, thanks. I have mentioned this in the new paragraph in the revised discussion.

Minor comments:

Comment R2.8.
Line 85-86: This should read the "former of which" should it not, as cooperative breeds would have been hypothesized to have a stronger response to inequity?

Response R2.8.
Yes! Thank you for catching this. I have made this change.

Comment R2.9.
Line 106: Should this read "…a relatively ¬lower payoff…" or something along these lines.

Response R2.9.
Thank you for this catch as well. I have edited.

Comment R2.10.

Lines 129 – 131: It seems as though the opening line of this paragraph is rehashing a point that has already been made. It is confusing on first reading as to why this point is brought up again as it has just been made already. It takes a while before it is clear what the author is getting at in the paragraph. Perhaps beginning this sentence with “although” or something similar, and restructuring the sentence accordingly would be helpful here.

Response R2.10.
Agreed. I revised the sentence so that it starts with although and then connects with a simplified version of the sentence referenced in Comment R2.11. The section now reads: “Although past work on dogs and wolves suggests that at least some social canids are averse to inequity—a response that may be tied to cooperation and the challenges that arise from it (e.g., food sharing, cooperative hunting, cooperative defense)—it also raises important questions.

Comment R2.11.
Lines 131 – 132: I find this sentence confusing: “However, past work also points to questions whose answers could help further bolster this claim.” I would consider revising and simplify it.

Response R2.11.
Agreed. I have edited this sentence to make it simpler.

Comment R2.12.
Line 137 and line 158: If the author wishes, the following reference could also be considered here: Oberliessen et al. 2016 Animal Behaviour ("Inequity aversion in rats"); it is not the same method as this study, but there are similarities particularly with the Horowitz 2012 dog study, as the subject had to make a choice related to the distribution of rewards between itself and a partner.

Response R2.12.
Thank you for drawing my attention to this paper, which I had not read. I now mention it in the discussion when discussing how these findings fit into the context of existing work on inequity aversion from across different paradigms.

Comment R2.13.
Lines 139 – 140: This line seems to be repetition of some of the information in lines 135 – 136. I understand from reading on that the author is setting up (in lines 135 onward) what will then be covered in the next 2 paragraphs but it does still come across as repetition. I would consider revising this slightly. Perhaps for example, lines 135-136 could state that the study compares dogs with a non-domesticated social canid….or in lines 139 onward beginning the paragraph with the assumption that the reader knows that Dingoes were tested in an inequity task?

Response R2.13.
I agree that this was repetitive and have implemented your suggested edit. Thank you for this suggestion.

Comment R2.14.
Line 152: Similar to the issue of repetition raised above, the point that “a novel task that requires no specific training” was already made, and was clear.

Response R2.14.
To avoid repetition I have made a similar edit to the one suggested in Comment R.13. I have shorted the description in the set-up section which now states “employing a novel task.” And I later elaborate on the point that the novel task required no specific training. I hope that removing this repetition has improved the flow of this section of the intro.

Comment R2.15.
Line 206: The use of the word confederate here and throughout the manuscript is unnecessary is it not? Wouldn't the word partner be sufficient?

Response R2.15.
Good point. I had used confederate to make it clear that these were dogs/dingoes who participated in multiple sessions unlike previous studies on inequity aversion. However, I agree that this is clear from the methods and supplementary table, so I have removed the word confederate and used partner except when

first introducing them.

Comment R2.16.
Line 332: What happened with the food if it was not placed on the plate? Did the experimenter hold it in their hand or place it in a pouch or something and if so, could the subject see this? Please clarify in the manuscript.

Response R2.16.
When calling the subject and partner, the food items were held on display in the air so that subjects could see them. The food items not placed on the plate were kept in the experimenters' hands and returned to the table that held the bowl of food along with the live coding notebook (Fig 1). The food items were small and were not being intentionally shown to subjects when they were returned to the table so my suspicion is that subjects could not see them.

Comment R2.17.
Lines 374 – 375: I do not understand the purpose of this sentence: "A video recording of each session was watched and analysed by a research assistant." The previous 4 paragraphs make it clear that the videos were analysed by research assistants. Perhaps this sentence refers to the reliability coding in which case I recommend combining it with the next sentence or rewording to make it clear that the reliability coding is being referred to here.

Response R2.17.
I removed this sentence.

Comment R2.18.
Line 377: To what does attention refer in this case?

Response R2.18.
This referred to food being presented in subjects' field of view. I have edited this section for clarity.

Comment R2.19.
Line 413: What is Protocol 28-25? Please provide a brief explanation e.g. is this from a university or government.

Response R2.19.
I now provide further information about IACUC approval.

Comment R2.20.
Line 420 – 424: There is no information given in the methods section on the structure of the models used for this analysis. Please include this.

Response R2.20.
I have now clarified this. Additionally, I have added the model output tables to the main text (now Tables 2 and 3; Note that I recreated these tables with bootstrapped CIs) which I hope will also help clarify the structure of the models. I have additionally provided the R code for analyses in addition to the raw data as supplemental material and will upload to OSF should the paper be accepted.

Comment R2.21.
Lines 442 – 443: What analysis is this conclusion based on specifically? Is this based on a non-significant main effect of the predictor session type or based on visual inspection of plots?

Response R2.21.
Apologies for confusion. I have clarified this in the text. The initial statement was a reference to the pattern of results revealed in Fig. 3(A). I have now made this clear and have given more details about the model associated with this research question and figure.

Comment R2.22.
Lines 579 – 580: What is meant here with inequity in non-social contexts? Does it refer to simply having two batches of food (or some other resource) that differ in quality or quantity? Please clarify this in the text.

Response R2.22.

I have edited this section for clarity.

Comment R2.23.
Lines 583 – 585: Please include a reference for dogs' referencing of humans when trying to solve a problem. See also Lazzaroni et al. 2020 Animal Cognition (why do dogs look back at a human in an impossible task); in sum, the results of this study suggest that much of dogs' looking to humans when attempting to solve difficult/impossible problems is simply a consequence of giving up with the task and looking at a salient stumulus.

Response R2.23.
I have made this change and now cite the Lazzaroni et al paper. Thank you for suggesting that reference.

Comment R2.24.
Line 598 – 599: The wording of this sentence seems to suggest that whether the partners in the previous studies were familiar or unfamiliar is an unknown. As this is not the intended meaning of the sentence, as far as I understand, consider rewording slightly to make it is clear that it is a possibility that the difference in use of the familiar vs unfamiliar partners between the studies is the reason for a difference in results.

Response R2.24.
Good point. I have edited this sentence for clarity.

Comment R2.25.
Line 609: Is there any research showing that dogs' attention differs based on familiarity? A reference would be particularly helpful here to support the point. See Horn et al. 2013 Animal Cognition ("Dogs' attention towards humans depends on their relationship, not only on social familiarity"); see also Range et al. 2009 Animal Cognition ("Social attention in keas, dogs, and human children"). But any insight from any species on the relationship between attention and familiarity would also be helpful here as a reference.

Response R2.25.
Thanks for raising this and for recommending those two papers. I have now added those citations to the discussion along with an additional paper by Mongillo et al.

Comment R2.26.
CSV files: It seems that there were different experimenters. It would be good to state this in the manuscript, including how many experimenters and whether the experimenter remained the same for a subject across conditions.

Response R2.26.
I now mention this in the revised MS under the Methods section.

Miscellaneous:

Comment R2.27.
Line 39: The use of square vs. round brackets differs from line 38.

Response R2.27.
Thanks! I fixed this.

Comment R2.28.
Line 66: Brosnan and de Waal should be the possessive i.e. Brosnan and de Waal's

Response R2.28.
Thanks! I fixed this.

Comment R2.29.
Line 556: This should read "current study" rather than "currents study".

Response R2.29.
Thanks! I fixed this.

Comment R2.30.

Table S4: Check use of big “X” for one of the interactions but small “x” for others.

Response R2.30.
I have used only small x’s in the revised SOM.

Comment R2.31.
Fig. S1, Fig. S2, and Fig. S3 legends: Here it states “…lines extending from boxes indicate minimum and maximum values and black circles indicate outliers”. The lines extending from the boxes do not seem to indicate the min. and max. values as there are additional values (the outliers) beyond their range.

Response R2.31.
Thanks for noting this source of potential confusion. These are calculated minima and maxima values and I have made this clear in the revised legends. Ggplot calculates the min and max values as Q1-1.5*IQR and Q3 + 1.5*IQR, respectively (where Q1 = first quartile, Q3 = third quartile and IQR = interquartile range). Outliers are any points outside of this range.
